# Correlated diffusion imaging

**DOI:** 10.1186/1471-2342-13-26

**Published:** 2013-08-08

**Authors:** Alexander Wong, Jeffrey Glaister, Andrew Cameron, Masoom Haider

**Affiliations:** 1 Department of Systems Design Engineering, U. of Waterloo, Waterloo, Canada; 2 Department of Medical Imaging, Sunnybrook Health Sciences Centre, Toronto, Canada

## Abstract

**Background:**

Prostate cancer is one of the leading causes of cancer death in the male population. Fortunately, the prognosis is excellent if detected at an early stage. Hence, the detection and localization of prostate cancer is crucial for diagnosis, as well as treatment via targeted focal therapy. New imaging techniques can potentially be invaluable tools for improving prostate cancer detection and localization.

**Methods:**

In this study, we introduce a new form of diffusion magnetic resonance imaging called correlated diffusion imaging, where the tissue being imaged is characterized by the joint correlation of diffusion signal attenuation across multiple gradient pulse strengths and timings. By taking into account signal attenuation at different water diffusion motion sensitivities, correlated diffusion imaging can provide improved delineation between cancerous tissue and healthy tissue when compared to existing diffusion imaging modalities.

**Results:**

Quantitative evaluation using receiver operating characteristic (ROC) curve analysis, tissue class separability analysis, and visual assessment by an expert radiologist were performed to study correlated diffusion imaging for the task of prostate cancer diagnosis. These results are compared with that obtained using T2-weighted imaging and standard diffusion imaging (via the apparent diffusion coefficient (ADC)). Experimental results suggest that correlated diffusion imaging provide improved delineation between healthy and cancerous tissue and may have potential as a diagnostic tool for cancer detection and localization in the prostate gland.

**Conclusions:**

A new form of diffusion magnetic resonance imaging called correlated diffusion imaging (CDI) was developed for the purpose of aiding radiologists in cancer detection and localization in the prostate gland. Preliminary results show CDI shows considerable promise as a diagnostic aid for radiologists in the detection and localization of prostate cancer.

## Background

Prostate cancer is the most common form of cancer diagnosed in men, with roughly 241,740 new cases in 2012 in the United States [1]. Furthermore, prostate cancer is the second leading cause of cancer death in males in the United States, with an estimated 28,170 deaths in 2012 [1]. Given that the median patient survival time for metastatic prostate cancer ranges from 12.2 to 21.7 months [2-6], early clinical diagnosis of prostate cancer is key to improve the treatment of patients affected by prostate cancer. Traditionally, clinical diagnosis of prostate cancer involves a prostate specific antigen (PSA) screening, where high PSA levels are considered indicative of possible signs of prostate cancer [7]. However, PSA screening has resulted in significant over-diagnosis of men suspected of having prostate cancer but who do not actually require treatment. As a consequence, many men are over-treated with therapies that carry significant risks in themselves [8]. Furthermore, there is still no reliable, widely accepted method of diagnostic imaging for prostate cancer. Although transrectal ultrasound (TRUS) is used routinely as a guide for biopsy, it cannot be used to visualize cancer foci because many tumours in the prostate gland are isoechoic and cannot be differentiated from surrounding tissue, resulting in sensitivity and specificity in the range of 40–50% [9,10]. Positron emission tomography (PET) have also been investigated as a potential imaging modality for prostate cancer detection, with a number of different tracers that have shown promise for identifying prostate cancer [11-14]. However, the spatial resolution achieved using PET may not be adequate to properly localize and detect early stage prostate cancer [15]. T2-weighted magnetic resonance imaging (MRI) has also been investigated for prostate cancer detection [16-18], but currently requires highly-qualified subspecialty radiologists to interpret the data due to its weak delineation between cancerous tissue and healthy tissue. Furthermore, in the peripheral zone of the prostate gland, the low T2 signal intensity that is associated with prostate cancer may also be due to a number of noncancerous abnormal conditions such as inflammation and hemorrhaging [19].

A promising imaging modality for diagnosing prostate cancer is diffusion imaging, where pairs of opposing magnetic field gradient pulses are applied to obtain sensitivity to the Brownian motion of water molecules in tissues [20]. The differences in diffusion characteristics between tissue types facilitate for tissue characterization. As such, given the presumed high cellular density of prostate cancer, the associated tissues should exhibit restricted diffusion characteristics (and as such should have lower apparent diffusion coefficient (ADC) values). While diffusion imaging shows considerable promise [21-23], particularly when used in multi-parametric imaging scenarios [24,25], delineating between cancerous tissue and healthy tissue in the prostate gland remains a challenge, due partly to the necessity for fine-tuning the strength, duration, and timing of the applied diffusion gradient pulses. Other challenges include the multifocality of prostate cancer [26], as well as the relatively small size of a majority of prostate cancer tumors. Hence, the characteristics between cancerous tissue and healthy tissue may appear to have substantial overlap depending on the way the gradient pulses are applied, thus making it difficult to detect and localize prostate cancer. As such, an alternative form of magnetic resonance imaging that gets around this issue is highly desired.

The main contribution of this study is the introduction of a new form of diffusion magnetic resonance imaging called correlated diffusion imaging (CDI), which takes advantage of the joint correlation in signal attenuation across multiple gradient pulse strengths and timings to not only reduce the dependency on the way diffusion gradient pulses are applied, but also improve delineation between cancerous and healthy tissue. To the best of the authors’ knowledge, there are no previous imaging techniques that take this type of approach to prostate cancer assessment.

## Methods

**Figure 1:**
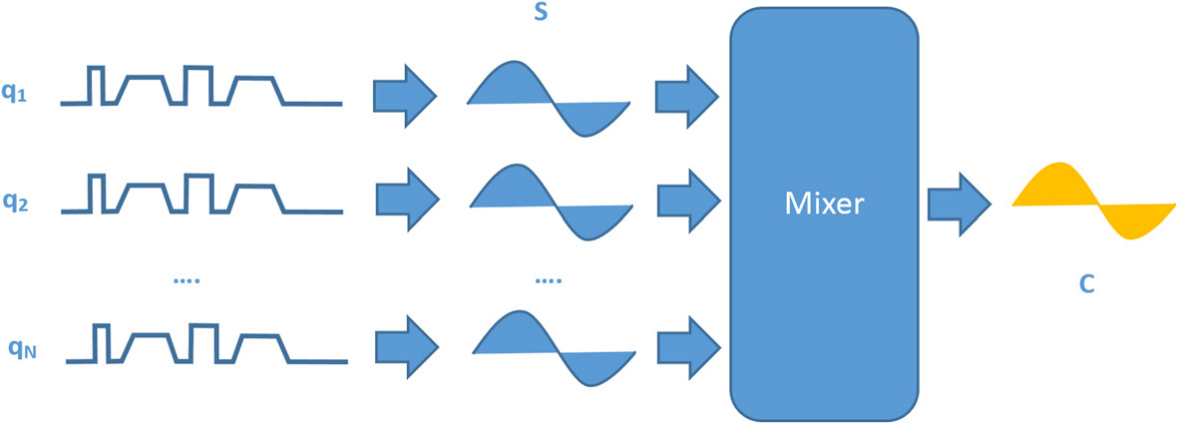
**The methodology behind correlated diffusion imaging.** The methodology behind correlated diffusion imaging (CDI) can be summarized as follows. First, multiple signal acquisitions are conducted using sequences with different gradient pulse strengths and timings (*q*_1_, *q*_2_, …, *q*_*N*_). Second, the acquired signals (*S*_1_, *S*_2_, …, *S*_*N*_) are then mixed together to obtain the local correlation of signal attenuation across the acquired signals, which produces a final signal (*C*) that characterizes the tissue being imaged.

The methodology behind correlated diffusion imaging (CDI) is summarized in Figure 1. First, multiple signal acquisitions are conducted at different gradient pulse strengths and timings. Second, the acquired signals are then mixed together to obtain the local correlation of signal attenuation across the acquired signals, which produces a final signal that characterizes the tissue being imaged. A detailed description of the steps involved is presented below.

### Imaging protocol

To evaluate the effectiveness of CDI for prostate cancer diagnosis, twenty patient cases with known prostate cancer were used in this study. The patients ranged in age from 58–80 years, with a median age of 69 years. Informed consent was obtained from all patients, and approval for this study was obtained from the ethics review board of Sunnybrook Health Sciences Centre. All results were reviewed by an expert radiologist with 16 years of experience interpreting body MRI and 11 years of experience interpreting prostate MRI.

Examinations using CDI were performed using a Philips Achieva 3.0T machine at Sunnybrook Health Sciences Centre, Toronto, Canada. The axial echo-planar sequence was performed for CDI with the following imaging parameters: TR range from 3336 – 6178 ms with a median of 4890 ms, and TE ranged from 61 – 67 ms with a median of 61 ms. The resolution of the signal acquisitions ranged from 1.36 × 1.36 mm^2^ to 1.67 × 1.67 mm^2^, with a median of 1.56 × 1.56 mm^2^. Slice thickness ranged from 3.0 – 4.0 mm with a median of 3.5 mm. The display field of view (DFOV) ranged from 20 × 20 cm^2^ to 24 × 24 cm^2^ with a median of 24 × 24 cm^2^.

For comparison purposes, apparent diffusion coefficient (ADC) maps were also obtained using the same axial echo-planar sequence with the same imaging parameters and Ω = {0*s*/*mm*^2^,100*s*/*mm*^2^,1000*s*/*mm*^2^}, as it is considered state-of-the-art for prostate cancer analysis in existing diffusion imaging [24]. Finally, axial T2-weighted imaging acquisitions with the same slice locations as the CDI sequence were obtained as a baseline reference of comparison. Examinations using T2-weighted imaging were performed using a Philips Achieva 3.0T machine with the following imaging parameters: TR range from 4688 – 7504 ms with a median of 6481 ms, and TE range from 110 – 120 ms with a median of 120 ms. Slice thickness ranged from 3.0 – 4.0 mm with a median of 3.5 mm. The display field of view (DFOV) ranged from 20 × 20 cm^2^ to 24 × 24 cm^2^ with a median of 24 × 24 cm^2^.

### Signal acquisition

**Figure 2:**
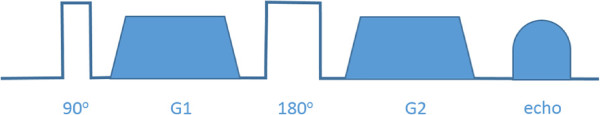
**Echo-planar sequence with two gradient pulses.** Echo-planar sequence with two gradient pulses (G1 and G2) of equal magnitude (one pulse in each side of the 180^*o*^ pulse to dephase and rephase the spins).

As the first step of the CDI imaging process, axial single-shot echo-planar sequences with two gradient pulses of equal magnitude (one pulse in each side of the 180^*o*^ pulse to dephase and rephrase the spins, respectively), as shown in Figure 2 are used to obtain multiple signal acquisitions using a set of different configurations of gradient pulse strengths and timings, which we will denote as Ω = {*q*_*i*_|*i* = 1,...,*N*}, where *q*_*i*_ denotes the *i*^th^ sequence.

Imperfect rephasing occurs due to motion of water molecules, leading to attenuation in the acquired signal and thus allowing for the study of water diffusion based on signal attenuation behavior. By varying the configuration of gradient pulse strengths and timings between signal acquisitions, each signal acquisition is sensitive to a different degree of Brownian motion of water molecules in tissues, thus providing unique information with respect to the water diffusion characteristics of the tissue being imaged. The different configurations of gradient pulse strengths and timings can be defined by the following set of parameters [27]:

(1) $$q_{i}=(G_{i},\delta_{i},\Delta_{i}),$$

where, for the *i*^th^ sequence, *G*_*i*_ denotes the gradient pulse strength, *δ*_*i*_ denotes the gradient pulse duration, and Δ_*i*_ denotes time between gradient pulses. By grouping the gradient terms, the configuration of gradient pulse strengths and timings used for a particular sequence *q*_*i*_ can be simplified to [28]

(2) $$q_{i}=\gamma^{2}G_{i}^{2}\delta_{i}^{2}(\Delta_{i}-\frac{\delta_{i}}{3}),$$

where *γ* denotes the proton gyromagnetic ratio.

### Signal mixing

As the second step of the CDI imaging process, the multiple signal acquisitions are mixed together to obtain the final signal that characterizes the tissue being imaged. Here, we are interested not in the signal attenuation obtained using the individual configurations of gradient pulse strengths and timings, but in the local correlation of signal attenuation **across** the different configurations of gradient pulse strengths and timings within a local spatial sub-volume *V* to provide a better overall characterization of the water diffusion properties of the tissue being imaged. As such, we would like to mix all of the signal acquisitions together into a single quantitative signal characterizing the local signal attenuation correlation.

To achieve this goal, we introduce the following signal mixing function $C(\underline{x})$ for characterizing local signal attenuation correlation, which is parameterized by diffusion range defined by [*q*_*α*_,*q*_*β*_] and is defined as

(3) $$\begin{array}{ll} \;C_{\left[q_{\alpha },q_{\beta }\right]}(\underline{x}) & =\int \ldots \int S_{q_{\alpha }}(\underline{x})\ldots S_{q_{\beta }}(\underline{x})f\left(S_{q_{\alpha }}(\underline{x}),\ldots ,S_{q_{\beta }}(\underline{x})|V(\underline{x})\right)\\ & \;\times {\text{dS}}_{q_{\alpha }}(\underline{x})\ldots {\text{dS}}_{q_{\beta }}(\underline{x}), \end{array}$$

where $\underline{x}$ denotes spatial location, *S* denotes the acquired signal, *f* denotes the conditional joint probability density function, and $V(\underline{x})$ denotes the local sub-volume around $\underline{x}$. For this study, [*q*_*α*_,*q*_*β*_] was set at [0*s*/*mm*^2^,2000*s*/*mm*^2^], and *V* was defined as a 7 mm^3^ spatial sub-volume for assessment purposes as it was found to provide good tissue delineation.

### Image analysis and interpretation

The ADC maps and CDI images were reconstructed using the ProCanVAS (Prostate Cancer Visualization and Analysis System) platform developed at the University of Waterloo Vision and Image Processing research group, and were analyzed such that each modality was analyzed independent of other modalities. All visual assessments were made by an expert radiologist with 16 years of experience interpreting body MRI and 11 years of experience interpreting prostate MRI.

### Statistical analysis

Two different analysis strategies were performed to quantify the potential of CDI as a tool for prostate cancer detection and localization. In the first analysis strategy, a receiver operating characteristic (ROC) curve analysis was performed using CDI to quantitatively assess prostate detection and localization. The ROC curves were estimated assuming bivariate normal data. For illustrative purposes, the ROC curves obtained from the pooled data of all patient cases was plotted. To provide a quantitative assessment of diagnostic accuracy, the area under the ROC curve (*A*_*z*_) was obtained as a single metric of diagnostic accuracy. For comparison purposes, ROC curve analysis was also performed using ADC map as the baseline reference method for assessing prostate cancer using diffusion imaging.

In the second analysis strategy, we wish to study whether CDI would be a useful imaging modality for building computer-aided clinical decision support systems to assist in the prostate cancer detection and localization process. To quantify the usefulness of CDI for the purpose of building such systems, leave-one-out cross-validation (LOOCV) trials were performed across all patient cases. For each trial, a two-class Maximum Likelihood (ML) classifier model is trained based on the CDI signal intensity statistics of the individual voxels within the prostate gland (one class characterizing cancerous tissue, with the other class characterizing healthy tissue) across the training patient cases. This learned two-class ML classifier model is then used to calculate sensitivity, specificity, and accuracy for the validation patient case. This process is repeated for a number of trials so that each patient case is used once as the validation patient case. The same was performed on ADC for comparative purposes.

## Results and discussion

**Figure 3:**
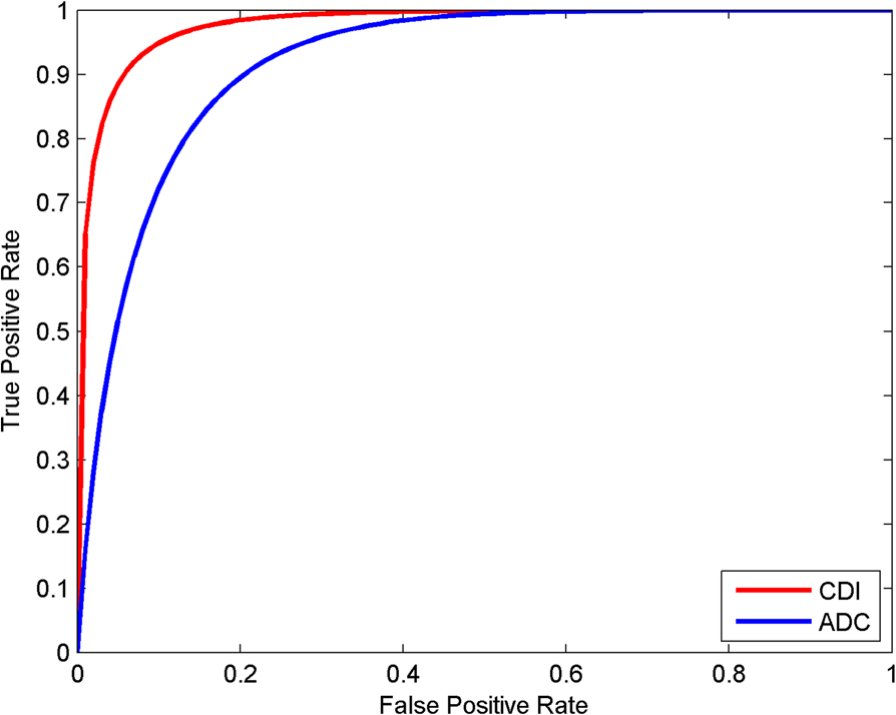
**Receiver operator characteristic (ROC) curves.** Receiver operator characteristic (ROC) curves from all patient cases for detection of prostate cancer using CDI (blue line) and ADC map (red line). For whole prostate, the area under the ROC curve (*A*_*z*_) was higher for CDI (*A*_*z*_ = 0.9789) than for ADC map (*A*_*z*_ = 0.9183).

To visualize the diagnostic performance for all patient cases, ROC curves for CDI and ADC map results from all patient cases are shown in Figure 3. It can be observed that improved ROC characteristics are exhibited by CDI when compared to ADC map. Furthermore, the area under the ROC curve for CDI is higher with *A*_*z*_ = 0.9789, compared to the ROC curve for ADC map with *A*_*z*_ = 0.9183. The overall sensitivity, specificity, and accuracy results from the LOOCV trials are shown in Table 1. It can be observed that the sensitivity, specificity, and accuracy are higher for CDI when compared to ADC, which indicates the potential usefulness of CDI as an imaging modality for building computer-aided clinical decision support systems to assist in the prostate cancer detection and localization process.

**Table 1:** Leave-one-out cross-validation (LOOCV) results

	**Sensitivity**	**Specificity**	**Accuracy**
CDI	0.8676	0.9444	0.9363
ADC	0.8236	0.7679	0.7691

**Figure 4:**
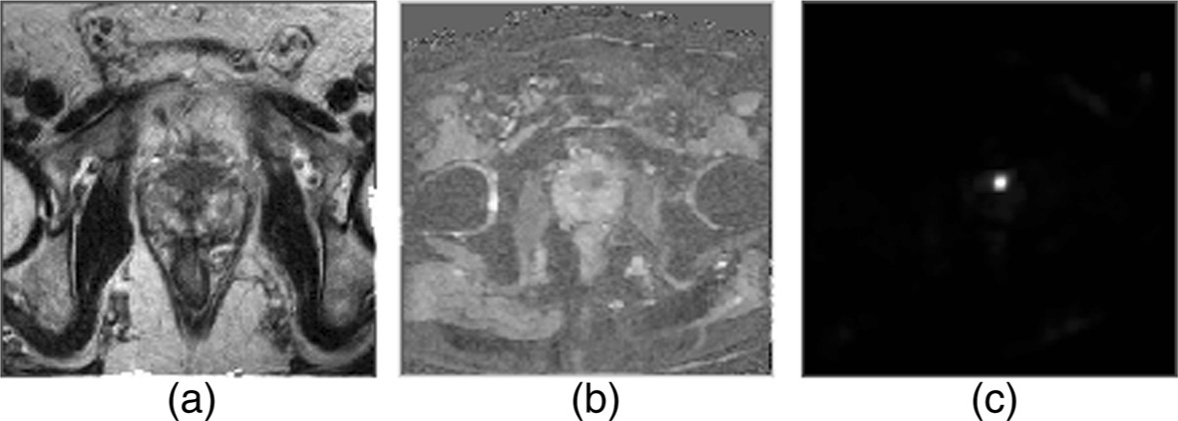
**Example case 1.** Tumor stands out well on CDI and not at all on T2-weighted imaging in patient with prostate cancer. **a)**, T2-weighted imaging shows no change in signal towards left side of transition zone. **b)**, ADC map shows increased contrast around left side of transition zone. **c)**, CDI shows very high signal intensity corresponding to left side of transition zone.

**Figure 5:**
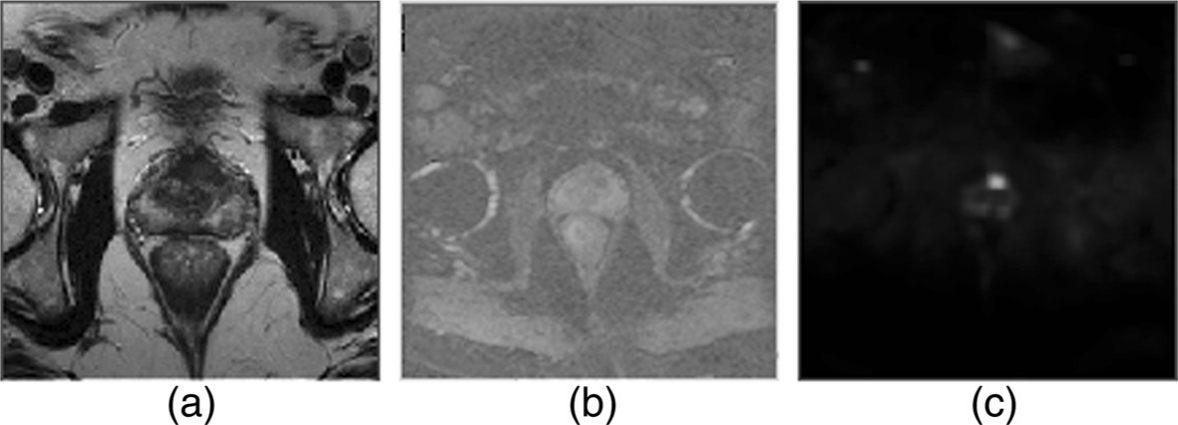
**Example case 2.** Tumor stands out well on CDI and not at all on T2-weighted imaging in patient with prostate cancer. **a)**, T2-weighted imaging shows no change in signal towards left side of transition zone. **b)**, ADC map shows increased contrast around left side of transition zone. **c)**, CDI shows very high signal intensity corresponding to left side of transition zone.

**Figure 6:**
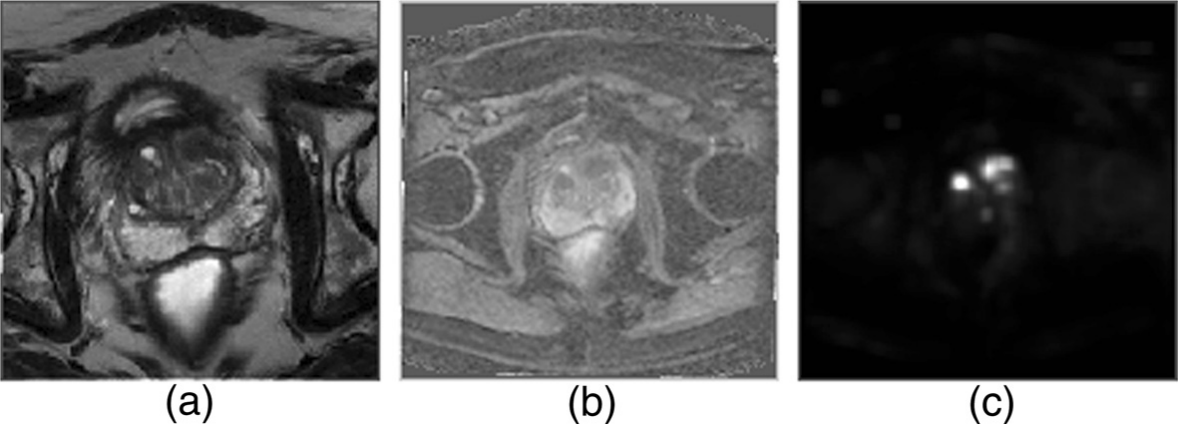
**Example case 3.** Tumors stand out well on CDI and poorly on T2-weighted imaging in patient with prostate cancer. **a)**, T2-weighted imaging shows mild decrease in signal towards left side of transition zone. **b)**, ADC map shows increased contrast around both sides of transition zone. **c)**, CDI shows very high signal intensity corresponding to both sides of transition zone.

Figures 4, 5 and 6 show example slices from T2-weighted imaging, ADC map, and CDI of five patient cases out of the twenty patient cases used in the ROC analysis, and a number of observations can be made. Note that example slices show cancerous regions within the prostate gland, not benign prostatic hyperplasia (BPH) nodules. There is weak visual delineation between prostate cancer and healthy tissue in the prostate gland in the T2-weighted imaging, thus making it difficult even for highly-qualified subspecialty radiologists to interpret (particularly in Figures 4 and 5 where there is no decrease in signal in the cancerous region). The ADC map provides improved visual delineation compared to the T2-weighted imaging; however, it can be observed that there are some cases (e.g., Figure 6) where the boundary delineation between tumor and healthy tissue is still difficult to assess. The CDI provides clearer indication of the locations and boundaries of the prostate cancer compared to the ADC maps for all patient cases. Hence, these preliminary results are motivating for the potential of CDI as a diagnostic tool for prostate cancer detection and localization.

## Discussion

In this study, we have introduced a new form of diffusion magnetic resonance imaging called correlated diffusion imaging, which quantifies joint correlation in signal attenuation across multiple diffusion gradient pulse strengths and timings. The preliminary results in this study show that CDI has potential to be an effective tool for prostate cancer detection and localization.

It is important to also understand the merits of CDI in relation to practical aspects of clinical image acquisition, post-processing, and analysis. One of the attractive characteristics of CDI from a clinical image acquisition perspective is that the signal acquisition process of CDI can be performed on existing clinical imaging systems without hardware modifications. The signal mixing and reconstruction process of CDI can all be performed post-acquisition on a computer workstation using additional computer-aided clinical decision support software such as the ProCanVAS (Prostate Cancer Visualization and Analysis System) platform developed at the University of Waterloo Vision and Image Processing research group. Therefore, the only additional resources needed for practical clinical imaging using CDI compared to other modalities is the need for software to reconstruct the CDI images. Once reconstructed, the CDI images can be viewed on any existing DICOM viewer software, making it easy to integrate into existing radiology workflows.

One possible explanation for CDI’s potential to be a more effective tool for prostate cancer detection and localization when compared to standard clinical practice ADC maps may be related to the highly restrictive water diffusion nature of prostate cancer. While different gradient pulse strengths and timings may be more sensitive to different degrees of water diffusion motion, this highly restrictive diffusion nature results in signal attenuation that is similar or lower than healthy tissue at all degrees of sensitivity. Therefore, this results in consistently low signal attenuation of prostate cancer compared with healthy tissue irrespective of gradient pulse strengths and timings which, in combination with the possible higher water content of cancerous tissue compared to healthy tissue [29], may lead to improved cancer and healthy tissue delineation in CDI.

A limitation of this study is the lack of substantial whole-mount histopathology as a reference standard to establish radiologic-pathologic correlation in a very comprehensive manner. Therefore, given these promising preliminary results, we suggest that CDI may be more thoroughly investigated for prostate cancer detection and localization, with a larger patient study that includes patient cases with known prostate cancer, healthy patient cases, and patient cases with benign prostatic hyperplasia (BPH) nodules (which can be mistaken for cancer under certain imaging modalities), an assessment of inter-observer variability, as well as comprehensive radiologic-pathologic correlation with a much larger set of prostate whole-mounts. Furthermore, given the potential of CDI as an effective tool for prostate cancer detection and localization, we suggest that studies be performed for other forms of cancers such as pancreatic cancer, breast cancer [30], and liver cancer [31], which may also show similar highly restricted water diffusion characteristics.

## Conclusions

In this study, a new form of diffusion magnetic resonance imaging called correlated diffusion imaging (CDI) was developed for the purpose of aiding radiologists in cancer detection and localization in the prostate gland. Preliminary results show CDI shows considerable promise as a diagnostic aid for radiologists in the detection and localization of prostate cancer. As such, given the promising results of this initial study, a future direction is to perform a larger, more comprehensive patient study to better evaluate the utility of CDI for the purpose of prostate cancer detection and localization.

## Competing interests

The authors have declared that no competing interests exist.

## Authors’ contributions

AW was involved in designing the methodology. AW, AC, and JG were involved in designing the study and performing statistical analysis. AW, AC, JG, and MH were involved in the writing and editing. MH was involved in collecting and reviewing the data. All authors reviewed the manuscript. All authors read and approved the final manuscript.

## Authors’ information

AW is an assistant professor in the Department of Systems Design Engineering at the University of Waterloo, Ontario, Canada, specializing in medical imaging. MH is the chief radiologist at Sunnybrook Health Sciences Centre, Toronto, Ontario, Canada, specializing in abdominal imaging, with particular expertise in prostate cancer imaging and assessment.

## Pre-publication history

The pre-publication history for this paper can be accessed here:

http://www.biomedcentral.com/1471-2342/13/26/prepub
